# Culturally Embedded Inner Strengths as Predictors of Resilience in Emerging Adults Following Childhood Parental Divorce

**DOI:** 10.3390/ejihpe16060073

**Published:** 2026-05-25

**Authors:** Shan Chen, Penkarn Kanjanarat, Tinakon Wongpakaran, Danny Wedding

**Affiliations:** 1 Mental Health Program, Multidisciplinary and Interdisciplinary School, Chiang Mai University, Chiang Mai 50200, Thailand; shan_c@cmu.ac.th (S.C.); danny.wedding@gmail.com (D.W.); 2 Department of Pharmaceutical Care, Faculty of Pharmacy, Chiang Mai University, Chiang Mai 50200, Thailand; 3 Department of Psychiatry, Faculty of Medicine, Chiang Mai University, Chiang Mai 50200, Thailand; 4 Department of Clinical and Humanistic Psychology, Saybrook University, Pasadena, CA 91103, USA; 5 Department of Psychology, University of Missouri-Saint Louis, St. Louis, MO 63121, USA

**Keywords:** parental divorce, emerging adulthood, resilience, culturally embedded inner strengths, equanimity, determination, loving-kindness, inner strengths, positive psychology

## Abstract

Parental divorce, as an adverse childhood experience, may disrupt family systems and exert enduring effects on psychological development, with impacts becoming particularly salient during emerging adulthood, a stage characterized by identity exploration and increasing autonomy. Within the Thai cultural context, inner strengths grounded in Buddhist values may serve as important resources for resilience. This study examined associations between inner strength dimensions derived from the Ten Perfections (*Pāramīs*) and resilience among emerging adults who experienced parental divorce in childhood. A cross-sectional design was employed with 160 Thai participants aged 20 to 29 years, who completed the Inner Strength-Based Inventory (I-SBI) and the Resilience Inventory (RI-9). Correlation analysis indicated that equanimity, determination, perseverance, wisdom, meditation, and loving-kindness were positively correlated with resilience. Multiple regression analysis showed that equanimity (β = 0.312, *p* < 0.001), determination (β = 0.227, *p* < 0.01) and loving-kindness (β = 0.213, *p* < 0.01) were significantly associated with resilience. These findings suggest that culturally embedded inner strengths are associated with resilience in a non-Western context. More specifically, among the ten dimensions examined, equanimity, determination, and loving-kindness emerged as being significantly associated with resilience in Thai emerging adults with childhood parental divorce experiences.

## 1. Introduction

Marriage institutionalizes intimate partnerships into structured family systems defined by shared rights and obligations, and childrearing responsibilities (Barber, 1974; Herden, 2024). With socioeconomic development and shifting relational norms, divorce has become an increasingly common means of dissolving marital dissatisfaction (Bennett et al., 2020; Bukhtiar et al., 2025; Cabılar & Yılmaz, 2022). Since the 1970s, global divorce rates have remained a prevalent feature of contemporary family life worldwide (Huang & Ma, 2024; Sheykhi, 2020). From the perspective of family systems theory, the family constitutes a dynamic whole composed of multiple interconnected subsystems (Bavelas & Segal, 1982). As the structural core of this system, the marital relationship is closely tied to the functioning of the entire family unit. Divorce may disrupt established interaction patterns and necessitates reorganization of roles, responsibilities, and emotional support networks, potentially generating enduring stress and psychological consequences for family members (Anderson, 2014; Bernet et al., 2016; Chavda & Nisarga, 2023; Eldar-Avidan et al., 2009).

In families with children, parental divorce is widely regarded as an adverse childhood experience, representing an abrupt and often incomprehensible life transition (Chipalo, 2025; Felitti et al., 1998; Turney, 2018). Parental divorce often places children in emotionally challenging situations, as parents prioritize their own needs over other responsibilities, forcing children to exert considerable effort to restore family balance and increasing their emotional insecurity (Garber, 2021). Younger children are especially vulnerable to anxiety, and prolonged parental conflict can intensify negative emotions and impair coping, with lasting developmental consequences in later life stages (Chavda & Nisarga, 2023; Damota, 2019). As individuals enter emerging adulthood (ages 18–29), a developmental stage characterized by identity exploration, relational commitment, and life-direction decisions (Arnett, 2007; Arnett et al., 2014; Silvers & Peris, 2023), earlier family experiences may resurface within new developmental demands (Dameron & Goeke-Morey, 2023; Presence, 2024). Internalized relational schemas formed in childhood may shape expectations about intimacy, emotion regulation, and coping with stress, thereby influencing adaptative functioning (Arnett, 2023; Eken, 2017; Newcomb-Anjo et al., 2018). For individuals who experienced parental divorce in childhood, early family disruption may have lasting implications for psychological adjustment and resilience as they navigate relational changes and developmental demands in emerging adulthood (Arnett et al., 2014; Bian & Ji, 2024; Kulka & Weingarten, 1979; Presence, 2024; Tullius et al., 2022).

Resilience is commonly understood as an individual’s dynamic capacity to develop within contexts characterized by both adversity and positive adaptation (Fletcher & Sarkar, 2013; Grygorenko & Naydonova, 2023; Métais et al., 2022). Its core lies in maintaining relatively stable psychological and physiological functioning through sustained internal psychological regulation and external coping strategies when facing stress, challenges, or significant life transitions (Fletcher & Sarkar, 2013; Nesbitt et al., 2022; Wu et al., 2013). Inner strengths support sustained adaptation and are associated with development of resilience, enabling more composed and resilient responses to life’s challenges (Norberg & Strandberg, 2025; T. Wongpakaran et al., 2023). Resilience is an important protective factor in adulthood, associated with reduced risk of depression and enhanced stress regulation (Franczok-Kuczmowska, 2022). Individuals with high resilience tend to recover more quickly from trauma, demonstrate greater adaptability, and show better emotional functioning, interpersonal relationships, and self-efficacy, thereby improving overall mental health and quality of life (Franczok-Kuczmowska, 2022; Kartika, 2017). Therefore, for emerging adults who experienced adverse childhood experiences, examining the factors associated with resilience is of particular importance (Luecken & Gress, 2010).

Since the mid-20th century, Thai society has gradually entered a transitional phase, with industrialization and urbanization driving changes in economic structure and social functioning. As educational opportunities expanded, women’s social status improved, and cultural contexts diversified, family organization and marital practices began to change (Limanonda, 1995; Panuwityo, 2025; Teerawichitchainan, 2004). Over the past decade, divorce in Thailand has trended upward. Official national statistics of Thailand reveal that between 2012 and 2024, the number of registered divorces increased from approximately 110,000 to nearly 150,000, representing an overall growth of 32.5% with an average annual growth rate of about 2.5% (National Statistical Office, 2025). The latest population data further indicates that by 2025, the stability of marital relationships in Thailand had declined, with approximately one in every three marriages ending in divorce. Meanwhile, the number of single mothers registered under the Newborn Child Support Grant scheme has also risen. In 2025, registrations increased by 7344 from the previous year, climbing from 120,978 to 128,322 (The Nation, 2025).

From a positive psychology perspective, the balanced cultivation and application of character strengths are associated with enhanced adaptive capacity and psychological well-being (Csikszentmihalyi & Seligman, 2000; Niemiec, 2023). Within the Thai cultural context, Buddhism occupies a central position in social life, profoundly shaping family values and individual psychological development (Limanonda, 1995). Inner strengths are often embodied through the Theravada Buddhist concept of the “Ten Perfections” (*Pāramīs*), which include truthfulness, perseverance, wisdom, generosity, the five precepts, meditation, tolerance, equanimity, determination, and loving-kindness. These virtues constitute the foundation of moral and psychological cultivation, and their sustained practice is believed to be associated with psychological transformation and higher levels of resilience (N. Wongpakaran et al., 2020; T. Wongpakaran et al., 2023). Prior research suggests substantial conceptual overlap between character strengths and inner strengths, as both represent essential internal psychological resources that individuals mobilize when encountering stress and life challenges (Norberg & Strandberg, 2025; N. Wongpakaran et al., 2020). Although inner strengths are conceptually informed by the Ten Perfections, they are can be conceptualized as universal positive psychological traits rather than religiosity-based constructs (N. Wongpakaran et al., 2020). Several dimensions of the Ten Perfections also align with other established constructs. For instance, equanimity reflects processes related to emotion regulation, including emotional stability and non-judgmental awareness (Raugh et al., 2025; Weber, 2021); determination resembles grit and goal-directed persistence (Ecker et al., 2024; Martin et al., 2025); loving-kindness corresponds to prosocial orientation, compassion, and goodwill (Lim et al., 2025; Petrovic et al., 2024; Ramkissoon, 2022); and meditation significantly overlaps with mindfulness (Gazder et al., 2025; Sünbül, 2020). These convergences further suggest that culturally embedded inner strengths may reflect contextually expressed forms of broader adaptive processes, providing a bridge between culture-specific constructs and resilience (Lee et al., 2025; Martin et al., 2025; Monteiro et al., 2021; Stagman-Tyrer, 2014; T. Wongpakaran et al., 2023).

Despite growing interest in resilience following parental divorce, little is known about how culturally embedded inner strengths are associated with resilience among Thai emerging adults who experienced parental divorce in childhood in non-Western contexts (Kulka & Weingarten, 1979; Sezer, 2025). The distinctiveness of Thai culture may be associated with variations in resilience among emerging adults following childhood parental divorce, although empirical evidence remains limited. To address this gap, the present study distinguishes ten dimensions of inner strength derived from the Ten Perfections (*Pāramīs*), enabling a more nuanced examination of their associations with resilience. The study also focuses on Thai emerging adults with a history of childhood parental divorce and provides evidence on culture-specific correlates of resilience. In addition, the study highlights the importance of integrating culturally embedded constructs into psychological research on resilience.

## 2. Materials and Methods

### 2.1. Participants and Data Collection

This cross-sectional study examined the relationship between culturally embedded inner strengths and resilience among Thai emerging adults who experienced childhood parental divorce. A total of 183 participants were recruited through online social media platforms (e.g., Facebook, TikTok, and Instagram) in January and February 2026.

Under Thai law, adulthood begins at age 20 (Civil and Commercial Code, 1925). Individuals aged 18–19 are legally minors with limited contractual capacity, even though they are no longer classified as “children” under the Child Protection Act (<18 years) (Child Protection Act, 2003). For this study, we restricted eligibility to participants aged 20 years and older to ensure that all respondents were legally recognized as adults and able to provide informed consent independently. Accordingly, the final sample comprised Thai emerging adults aged 20 to 29 years who reported awareness of the legal divorce of their biological parents during childhood (before the age of 18) and were able to identify the age at which the parental divorce occurred. All participants were required to be sufficiently proficient in Thai to understand and accurately complete the questionnaire. Individuals were excluded if they had been previously diagnosed with psychological disorders (e.g., depression, schizophrenia, bipolar disorder, or post-traumatic stress disorder), were currently undergoing psychotherapy or receiving psychiatric medication, or had cognitive impairments that might interfere with valid responding. Participation was voluntary, and informed consent was obtained electronically prior to survey completion.

### 2.2. Sample Size and Data Screening

Of 183 responses received, 16 incomplete and 4 ineligible responses were excluded, yielding 163 valid participants. After data screening and the removal of three outliers, the final analytic sample comprised 160 participants. The target sample size was determined based on the guideline for multiple regression proposed by Green (1991) (N ≥ 104 + k, where *k* represents the number of predictors). With 17 predictors, the minimum required sample size was 121 to detect medium-sized effects at α = 0.05 and power = 0.80. The final sample size exceeded this requirement, indicating adequate power for detecting medium-sized effects.

### 2.3. Model Specification and Analytical Justification

The regression model was specified *a priori* to balance theoretical completeness with parsimony. Sociodemographic variables were included as covariates due to their documented associations with resilience and related adjustment outcomes (Angela et al., 2024; Arrastía-Chisholm et al., 2025; Hanımoğlu, 2025; Laird et al., 2020; Na et al., 2022; Shin et al., 2010; van den Berg & Verbakel, 2024), as well as to account for their potential to confound the associations between inner strengths and resilience. The primary predictors were the ten dimensions of inner strength from the Buddhist Ten Perfections (*Pāramīs*) framework (N. Wongpakaran et al., 2020). All ten dimensions were entered simultaneously to preserve theoretical fidelity and full construct coverage, consistent with theory-driven modeling strategies.

To further justify sample adequacy for this prespecified model, a sensitivity power analysis was conducted using G*Power version 3.1.9.7 (F tests; linear multiple regression, fixed model, R^2^ deviation from zero; α = 0.05; 17 predictors; N = 160). The results indicated that the sample provided 80% power (1 − β = 0.80) to detect an overall effect size of f^2^ = 0.135, corresponding to R^2^ ≈ 0.119. This indicates adequate statistical power to detect small-to-moderate effects within the prespecified regression model.

### 2.4. Instruments

#### 2.4.1. Sociodemographic Information

Participants provided sociodemographic information including age, gender, education level, monthly income, age at the time of parental divorce, primary caregiver after parental divorce and current marital status. These variables were used to describe the sample and, where appropriate, were included as covariates in subsequent analyses.

#### 2.4.2. Inner Strength-Based Inventory (I-SBI)

Culturally embedded inner strengths were assessed using the Inner Strength-Based Inventory (I-SBI). The I-SBI was developed based on the concept of the Ten Perfections in Theravada Buddhism and consists of 10 items, each representing a distinct dimension of inner strength: truthfulness, perseverance, wisdom, generosity, observance of the five precepts, meditation, tolerance, equanimity, determination, and loving-kindness. Each item presents five descriptive statements reflecting increasing levels of the trait, and participants were instructed to select the option that best described them. Responses were scored on a five-point scale from 1 to 5, with higher scores indicating greater levels of inner strength. The I-SBI assesses these ten dimensions as universal character strengths rather than as indicators of religiosity or Buddhist devotional commitment. Rasch analysis demonstrated satisfactory measurement reliability, with a person separation reliability coefficient of 0.86, indicating adequate consistency in distinguishing respondents, and an item reliability coefficient of 0.99, suggesting a stable item hierarchy (N. Wongpakaran et al., 2020). In the present study, all items were included.

#### 2.4.3. Resilience Inventory (RI-9)

Resilience was measured using the Resilience Inventory (RI-9), an instrument grounded in the theoretical framework of inner strength. Rather than conceptualizing resilience solely as stress recovery, the RI-9 frames it as a multidimensional construct encompassing perseverance, wisdom, patience, mindfulness, loving-kindness, and equanimity. The scale contains nine items; each rated on a five-point Likert scale ranging from 1 (“does not describe me at all”) to 5 (“describes me very well”). Higher scores indicate greater resilience. Previous psychometric evaluation using the Rasch model reported a person reliability coefficient of 0.86, indicating adequate measurement precision. The original validation study also demonstrated strong internal consistency, with a Cronbach’s alpha of 0.90 (T. Wongpakaran et al., 2023). In the current sample, the RI-9 showed good internal consistency, with a Cronbach’s alpha of 0.87.

### 2.5. Statistical Analyses

All statistical analyses were conducted using IBM SPSS Statistics version 27.0. Descriptive statistics were computed to summarize participants’ sociodemographic characteristics and variables, including means, standard deviations, frequencies, and percentages. Reliability of the instruments was assessed using Cronbach’s alpha coefficients. Relationships among inner strengths, resilience and sociodemographic variables were evaluated using correlation analysis.

To examine the extent to which inner strength dimensions were associated with resilience, a multiple regression analysis was conducted. Prior to multiple linear regression, underlying assumptions were tested. Linearity and homoscedasticity were examined using a scatterplot of standardized residuals versus standardized predicted values. Normality of residuals was assessed using histograms and Normal P–P plots. Independence of errors was examined using the Durbin–Watson statistic, with values between 1.5 and 2.5 indicating no autocorrelation. Multicollinearity was assessed using Tolerance (values < 0.10 indicating concern) and Variance Inflation Factor (VIF; values > 5 indicating concern). Outliers and influential cases were identified based on standardized residuals (±3), Mahalanobis distance (*p* < 0.001), and Cook’s distance (>1.0). In the multiple regression analysis, resilience was entered as the dependent variable, inner strength dimensions as predictors, and age, gender, educational level, monthly income, age at parental divorce, primary caregiver after parental divorce, and current marital status as covariates. A significance level of 0.05 was applied for all statistical tests (Meyers et al., 2013).

## 3. Results

### 3.1. Descriptive Statistics

The study included 160 Thai emerging adults aged 20–29, predominantly female. Other demographic information, including educational level, monthly income, age at parental divorce, primary caregiver after parental divorce, and current marital status, is presented in Table 1.

Descriptive statistics are presented in Table 2. The total inner strength score ranged from 22 to 42 (Mean = 31.55, SD = 4.45). Mean scores for the ten dimensions ranged from 1.68 to 3.79, with generosity highest and meditation lowest. The overall resilience score ranged from 18 to 45 (Mean = 35.24, SD = 6.10).

### 3.2. Correlation Analysis

Correlation coefficients are presented in Table 3. Equanimity, determination, loving-kindness, perseverance, wisdom, and meditation were positively associated with resilience (*p* < 0.05), whereas truthfulness, generosity, the five precepts, and tolerance were not. Older age and female gender were associated with higher resilience.

### 3.3. Multiple Regression Analysis

A multiple regression analysis was conducted to examine the associations between inner strength dimensions, sociodemographic variables, and resilience (Table 4).

Scatterplots of the residuals supported the assumptions of linearity and homoscedasticity. The histogram and Normal P–P plot indicated that the residuals were approximately normally distributed. The Durbin–Watson statistic was 1.924, suggesting no autocorrelation was present. Tolerance values ranged from 0.681 to 0.898 and VIF values ranged from 1.113 to 1.469, indicating no multicollinearity. Potential outliers were evaluated based on standardized residuals, which did not exceed ±3 (maximum = 2.079). Cook’s distance values were all below 1 (maximum = 0.052), indicating that no cases exerted undue influence on the model. In addition, the maximum Mahalanobis distance was 34.944, which did not exceed the critical χ^2^ value at *p* < 0.001 for 17 degrees of freedom, indicating the absence of multivariate outliers. The overall model was statistically significant, F (17, 142) = 5.362, *p* < 0.001, accounting for a substantial proportion of the variance in resilience (R^2^ = 0.391, adjusted R^2^ = 0.318).

Among the inner strength dimensions, equanimity (β = 0.312, *p* < 0.001), determination (β = 0.227, *p* < 0.01), and loving-kindness (β = 0.213, *p* < 0.01) showed significant positive associations with resilience. Truthfulness (β = −0.088, *p* = 0.222), perseverance (β = 0.093, *p* = 0.213), wisdom (β = 0.113, *p* = 0.105), generosity (β = 0.070, *p* = 0.338), the five precepts (β = −0.027, *p* = 0.705), meditation (β = 0.041, *p* = 0.565), and tolerance (β = −0.021, *p* = 0.769) were not significantly associated with resilience.

Among the sociodemographic variables, age (β = 0.272, *p* < 0.001) and gender (female) (β = 0.290, *p* < 0.001) were significantly associated with resilience, whereas the remaining sociodemographic variables were not.

## 4. Discussion

This study examined the associations between inner strength dimensions derived from the Ten Perfections and resilience among Thai emerging adults who experienced parental divorce in childhood. Three dimensions of inner strength (equanimity, determination, and loving-kindness) were positively associated with resilience after controlling for sociodemographic variables. These findings suggest that culturally embedded inner strengths are associated with resilience in this context, although the cross-sectional design precludes causal interpretation.

The results are consistent with prior research on emerging adults (Dobles Villegas et al., 2025) and evidence that adolescents from divorced families can maintain relatively high resilience (Aryani et al., 2023). The Ordinary Magic Framework (Masten, 2001, 2025) conceptualizes resilience as emerging from normative adaptive systems rather than exceptional traits. Consistent with this view, the present findings suggest that specific dimensions of inner strength are differentially associated with resilience; these associations may involve adaptive coping processes such as self-awareness, emotional balance, sustained goal-directed effort, and compassion (Buddhaghosa, 2020; Thavaro, 1982; T. Wongpakaran et al., 2023). However, direct measurement of self-regulatory processes is needed to empirically test these potential mechanisms.

Equanimity reflects emotional balance and reduced reactivity, characterized by the capacity to remain stable across pleasant and unpleasant experiences (Chems-Maarif et al., 2025; N. Wongpakaran et al., 2020). In the present sample, higher equanimity was associated with higher resilience. Conceptually, equanimity overlaps with adaptive emotion regulation strategies such as acceptance and non-judgmental awareness (Chems-Maarif et al., 2025; Desbordes et al., 2015; Polizzi & Lynn, 2021). Converging empirical evidence from a recent meta-analysis confirms that such adaptive strategies are strongly associated with resilience (Raugh et al., 2025). Reduced emotional reactivity may help individuals sustain cognitive clarity and constructive coping under stress (Chan et al., 2014; Morse et al., 2021). These convergences suggest that equanimity, although rooted in the Ten Perfections (*Pāramīs*), may reflect general adaptive regulatory processes relevant across cultural contexts (Masten, 2001; Raugh et al., 2025; Terrana & Al-Delaimy, 2023). Longitudinal or experimental studies are required to further examine this pathway.

The dimension of determination, derived from the Ten Perfections (*Pāramīs*), reflects an individual’s tendency to persist in pursuing meaningful goals despite obstacles and setbacks (N. Wongpakaran et al., 2020). Conceptually, determination overlaps with established psychological constructs such as grit and perseverance, which emphasize sustained effort and long-term goal commitment in the face of adversity (Duckworth et al., 2007; Ecker et al., 2024; Peterson & Seligman, 2004), and these constructs have been associated with resilience (Martin et al., 2025). The Goal-Directed Resilience in Training (GRIT) model posits that sustained goal engagement is associated with resilience-related capacities, including persistence and adaptive coping (Kent et al., 2015). More broadly, engagement in meaningful long-term goals has been proposed to be associated with adaptive responses to environmental demands (Biggs et al., 2024; Danley, 2025). In emerging adulthood, goal-directed persistence has also been linked to resilience alongside factors such as sense of purpose, identity formation, and sustained effort (Danley, 2025). Nevertheless, these interpretations should be considered cautiously given the cross-sectional design of the present study.

Loving-kindness (*mettā)*, one of the Ten Perfections (*Pāramīs*), is also classified as one of the four sublime abodes *(Brahmavihāras*), alongside compassion (*karuṇā*), sympathetic joy (*muditā*), and equanimity (*upekkhā*) (Thavaro, 1982). It denotes a quality of unconditional goodwill and benevolence cultivated through meditation and daily practice (Salzberg & Kabat-Zinn, 2004; Thepa, 2022; N. Wongpakaran et al., 2020). In the present study, higher levels of loving-kindness were associated with greater resilience. This finding aligns with previous research indicating that compassion, empathy, and social connectedness are associated with adaptive psychological functioning and prosocial behavior (Hart, 2024; Quirk & Ivtzan, 2017; Seppala et al., 2014; Yin & Wang, 2023). Moreover, prosocial behaviors, such as helping others, volunteering, or supporting vulnerable groups, have been associated with positive emotions, increased social connectedness, and resilience (Moore et al., 2023; Wan et al., 2023). Future studies could test whether cultivating *mettā* (e.g., through structured practice) is associated with changes in resilience over time, beyond dispositional tendencies, using longitudinal designs or intervention-based approaches.

In the full model, several inner strength dimensions did not show significant associations with resilience. As is common in multiple regression analyses, non-significant associations may reflect overlap with stronger predictors or indirect associations. Some dimensions might also be more strongly related to other psychological outcomes, such as self-harm, depression, self-esteem, or perceived stress, rather than resilience (DeMaranville et al., 2025; Sripunya et al., 2024; N. Wongpakaran et al., 2025). Mediation analyses are needed to examine potential indirect associations.

Among sociodemographic variables, age and gender were significantly associated with resilience. Age showed a positive association with resilience, consistent with prior research suggesting that resilience is positively associated with age, possibly reflecting accumulated life experience, improved emotion regulation, and problem-solving abilities (Fletcher & Sarkar, 2013; Na et al., 2022; Naseem & Munaf, 2020; Rutter, 2012). Females reported higher resilience than males in this sample, which may reflect gender differences in coping and socialization, with females more likely to rely on social support and relational coping strategies associated with higher resilience (Blatt-Eisengart et al., 2009; Hanımoğlu, 2025; Lakomý & Kafková, 2017). Other sociodemographic variables were not significantly associated with resilience. However, nonsignificant findings should be interpreted with caution, as regression coefficients may be influenced by shared variance among predictors and sample composition, even when the overall model has adequate statistical power.

Taken together, the findings align with a risk–protective framework (Fraser et al., 1999; Pedro-Carroll, 2005), but the cross-sectional design precludes causal or directional conclusions. Longitudinal research is needed to clarify temporal relationships.

### 4.1. Implications

*Theoretical.* The findings suggest that inner strengths are differentially associated with resilience in the multivariable model. Further, these culturally embedded inner strengths show conceptual overlap with other established constructs, thereby contributing to theoretical integration. They may represent contextually expressed forms of general adaptive processes, linking culturally embedded psychological constructs with resilience. However, given the cross-sectional design, the direction of associations cannot be determined, and the findings should be interpreted strictly as associations rather than evidence of causal pathways. In addition, it remains unclear whether the associations with resilience reflect shared or culture-specific mechanisms.

*Cultural.* The results provide preliminary evidence that strengths conceptually grounded in the Buddhist Ten Perfections are associated with resilience among Thai emerging adults with a history of childhood parental divorce. This highlights the value of culturally embedded constructs in resilience research, while underscoring the need for replication across samples and for comparative work in both Buddhist-influenced and non-Buddhist cultural contexts to evaluate generalizability.

*Practical.* Although causal interpretations are not warranted, the identified dimensions (e.g., equanimity, determination, and loving-kindness) may inform future prevention and intervention research. Programmatic work could examine whether mindfulness- and meditation-based approaches (Juneau et al., 2020; Weber, 2021), goal-directed training (Kent et al., 2015), and loving-kindness practices or prosocial activities (Moore et al., 2023; Seppala et al., 2014) are associated with these dimensions and resilience. However, evidence from Thai samples suggests that the potential benefits of loving-kindness may depend on individual personality traits, highlighting the need to consider trait-based subgroup differences when applying loving-kindness-based approaches to support resilience (Phyu et al., 2026). Such approaches should be evaluated using longitudinal and experimental designs before recommending broad implementation.

### 4.2. Limitations and Future Study

This study has several limitations that warrant careful interpretation. First, the cross-sectional design and use of multiple regression preclude definitive conclusions about causal relationships. Future research should use longitudinal or experimental designs to clarify the direction of associations.

Second, although the overall model was statistically significant, the sample size relative to the number of predictors raises concerns about overfitting and coefficient stability, potentially limiting generalizability. In addition, the relatively short recruitment period may have restricted sample diversity. Non-significant predictors in this model may reflect weak unique associations or shared variance; therefore, these findings should be interpreted with caution. Future studies should employ larger, more diverse samples and use parsimonious, better-fitting models.

Third, reliance on self-report measures may introduce bias, including recall bias, social desirability, or common method variance, suggesting a need for objective or multi-informant measures in future research.

Finally, the sample of Thai emerging adults and the culturally embedded inner strengths may limit generalizability, highlighting the need for cross-cultural replication. The lack of a comparison group without parental divorce also limits interpretation regarding specificity to adverse experiences. Although the Inner Strength-Based Inventory assesses broadly relevant strengths, some dimensions may still be influenced by religiosity (e.g., Buddhist practice frequency, meditation practice, religious commitment), which was not measured in this study. Therefore, future study should include religiosity indicators and ideally adopt comparison group and/or longitudinal designs to clarify these issues.

## 5. Conclusions

This study examined culturally embedded inner strengths derived from the Ten Perfections (*Pāramīs*) in relation to resilience among Thai emerging adults with a history of childhood parental divorce. Among the ten dimensions of inner strength examined, equanimity, determination, and loving-kindness were positively associated with resilience. These results highlight the relevance of culturally embedded inner strengths as correlates of resilience in non-Western contexts. While no causal inferences can be drawn from the present cross-sectional design, interventions that foster emotional balance, goal-directed engagement, and empathy and compassion could be explored in future intervention research. Future experimental or longitudinal studies are needed to determine whether changes in these inner strength dimensions are associated with corresponding changes in resilience.

## Figures and Tables

**Table 1:** Sociodemographic information and characteristics (*n* = 160).

Variable	*n* (%)
Age (years)	
Mean ± SD	24.14 ± 2.999
Gender	
Female	134 (83.8)
Male	26 (16.3)
Educational level	
Lower secondary school	6 (3.8)
Upper secondary school	17 (10.6)
Bachelor’s degree or higher	137 (85.6)
Monthly income	
Less than 15,000 THB	82 (51.2)
15,000–35,000 THB	72 (45.0)
More than 35,000 THB	6 (3.8)
Age at the time of parental divorce (years)	
Up to 12	122 (76.3)
13–18	38 (23.8)
Primary caregiver after parental divorce	
Mother	78 (48.8)
Father	31 (19.4)
Grand parents	34 (21.3)
Other relatives	12 (7.5)
Other *	5 (3.1)
Current marital status	
Single	92 (57.5)
Dating/In a relationship	58 (36.3)
Married	5 (3.1)
Divorced	3 (1.9)
Other	2 (1.3)

Note. * Includes non-family caregivers, mixed or alternating arrangements, self-care, and other types of caregivers.

**Table 2:** Descriptive characteristics of the variables (*n* = 160).

Variable	Min.	Max.	Mean ± SD	Skewness	Kurtosis
Truthfulness	1	5	3.33 ± 1.09	−0.014	−1.270
Perseverance	1	5	2.64 ± 1.00	0.804	−0.337
Wisdom	1	5	3.43 ± 1.04	−0.302	−0.344
Generosity	1	5	3.79 ± 0.97	−0.885	−0.038
Five Precepts	1	5	3.18 ± 1.24	−0.133	−0.977
Meditation	1	5	1.68 ± 0.78	1.512	3.577
Tolerance	1	5	3.35 ± 1.00	−0.071	−1.033
Equanimity	1	5	3.28 ± 1.04	−0.301	−0.668
Determination	1	5	3.36 ± 1.06	−0.217	−0.813
Loving-Kindness	1	5	3.52 ± 1.16	−0.499	−0.901
I-SBI Total	22	42	31.56 ± 4.45	0.322	−0.355
RI Total	18	45	35.24 ± 6.10	−0.627	0.111

Note. *n* = number of participants; SD = standard deviation; I-SBI = Inner Strength-Based Inventory; RI = Resilience Inventory.

**Table 3:** Correlations Among Variables (*n* = 160).

Variable	1	2	3	4	5	6	7	8	9	10	11	12	13	14	15	16	17	18
1-Age	1																	
2-Gender (female)	−0.04	1																
3-Education (bachelor or higher)	0.04	0.11	1															
4-Income (<15,000 THB)	−0.43 **	0.01	−0.11	1														
5-Age at parental divorce (up to 12)	−0.07	0.07	0.02	−0.07	1													
6-Primary caregiver (mother)	0.02	0.06	−0.03	−0.02	0.05	1												
7-Marital status (single)	−0.15	−0.00	0.12	0.07	−0.06	0.05	1											
8-Truthfulness	0.15	−0.04	0.16 *	−0.31 **	0.09	−0.03	0.04	1										
9-Perseverance	0.10	−0.09	−0.08	−0.10	−0.17 *	0.09	−0.05	0.07	1									
10-Wisdom	0.02	−0.05	0.05	−0.02	0.01	−0.04	0.04	0.12	0.20 **	1								
11-Generosity	−0.05	−0.15	0.09	−0.02	0.03	−0.04	0.01	0.07	0.05	−0.01	1							
12-Five Precepts	−0.07	−0.06	0.03	0.13	−0.11	−0.07	0.11	0.04	0.19 *	0.08	0.13	1						
13-Meditation	0.10	−0.18 *	0.04	−0.09	−0.06	−0.05	0.07	−0.05	0.21 **	0.13	0.06	0.12	1					
14-Tolerance	−0.11	0.02	0.09	0.05	0.05	−0.12	−0.02	0.14	0.03	0.14	0.05	0.22 **	−0.01	1				
15-Equanimity	−0.07	−0.13	0.14	0.04	−0.12	−0.02	−0.16	0.06	0.16 *	0.09	0.02	0.04	0.08	0.16 *	1			
16-Determination	0.06	−0.12	−0.06	−0.04	−0.15	0.00	−0.13	−0.05	0.27 **	0.18 *	0.03	0.06	0.21 **	−0.06	0.04	1		
17-Loving-Kindness	−0.08	−0.05	0.14	0.17 *	−0.07	0.17 *	−0.03	−0.03	0.10	0.12	0.35 **	0.09	0.08	0.14	−0.12	0.14	1	
18-Resilience	0.21 **	0.17 *	0.13	−0.02	−0.14	−0.03	0.10	−0.04	0.23 **	0.22 **	0.10	0.44	0.16 *	0.04	0.29 **	0.29 **	0.21 **	1

Note. *n* = number of participants * *p* < 0.05 ** *p* < 0.01.

**Table 4:** Multiple Regression Analysis of Factors Associated with Resilience (*n* = 160).

Variable	B	SE	β	t	*p* Value	Tolerance	VIF
Constant	−0.68	5.505	—	−0.123	0.902	—	—
Truthfulness	−0.494	0.403	−0.088	−1.226	0.222	0.826	1.211
Perseverance	0.566	0.452	0.093	1.252	0.213	0.783	1.277
Wisdom	0.663	0.407	0.113	1.630	0.105	0.887	1.128
Generosity	0.437	0.454	0.070	0.962	0.338	0.815	1.226
Five Precepts	−0.131	0.347	−0.027	−0.379	0.705	0.852	1.174
Meditation	0.319	0.553	0.041	0.577	0.565	0.858	1.165
Tolerance	−0.128	0.435	−0.021	−0.294	0.769	0.836	1.196
Equanimity	1.832	0.423	0.312 ***	4.328	<0.001	0.824	1.213
Determination	1.302	0.411	0.227 **	3.167	0.002	0.832	1.203
Loving-Kindness	1.122	0.412	0.213 **	2.722	0.007	0.699	1.431
Age	0.555	0.152	0.272	3.643	<0.001	0.767	1.305
Gender (female)	4.788	1.139	0.290	4.202	<0.001	0.898	1.113
Educational level (bachelor’s degree or higher)	0.425	1.224	0.025	0.348	0.729	0.861	1.161
Monthly income (<15,000 THB)	0.477	0.966	0.039	0.494	0.622	0.681	1.469
Age at the time of parental divorce (up to 12 years)	−0.334	0.989	−0.023	−0.338	0.736	0.896	1.116
Primary caregiver after parental divorce (mother)	−1.121	0.841	−0.092	−1.333	0.185	0.898	1.114
Current marital status (single)	1.616	0.857	0.131	1.886	0.061	0.884	1.131
R^2^	0.391						
Adjusted R^2^	0.318						
F Test	F (17, 142) = 5.362, *p* < 0.001						

Note. Dependent Variable = Resilience; B = unstandardized coefficient; SE = standard error; β = standardized coefficient; R^2^ = coefficient of determination; Adjusted R^2^ = adjusted coefficient of determination; VIF = variance inflation factor; *n* = number of participants. ** *p* < 0.01 *** *p* < 0.001.

## Data Availability

The data supporting the findings of this study are available from the corresponding author upon reasonable request.

## Abbreviations

The following abbreviations are used in this manuscript:

I-SBI	Inner Strength-Based Inventory
RI	Resilience Inventory
VIF	Variance Inflation Factor
GRIT	Goal-Directed Resilience in Training
